# Study design to evaluate a group-based therapy for support persons of adults on buprenorphine/naloxone

**DOI:** 10.1186/s13722-020-00199-2

**Published:** 2020-07-11

**Authors:** Karen Chan Osilla, Kirsten Becker, Liisa Ecola, Brian Hurley, Jennifer K. Manuel, Allison Ober, Susan M. Paddock, Katherine E. Watkins

**Affiliations:** 1 RAND Corporation, 1776 Main Street, P.O. Box 2138, Santa Monica, CA 90407-2138 USA; 2 RAND Corporation, 1100 South Hayes Street, VA 22202 Arlington, USA; 3 LA County Department of Health Services, UCLA Department of Family Medicine, 10780 Santa Monica Blvd., Suite 105, Los Angeles, CA 90025 USA; 4 Department of Psychiatry, University of California San Francisco, 401 Parnassus Ave, San Francisco, CA 94143 USA; 5 San Francisco VA Health Care System, 4150 Clement St, San Francisco, CA 94121 USA; 6 NORC at the University of Chicago, 55 East Monroe St, 31st Floor, Chicago, IL 60603 USA

**Keywords:** Opioid use disorders, Medication assisted treatment, CRAFT, Family, Buprenorphine

## Abstract

**Background:**

Opioid use disorders (OUDs) have devastating effects on individuals, families, and communities. While medication treatments for OUD save lives and are increasingly utilized, rates of treatment dropout are very high. In addition, most existing medication treatments for OUD may often neglect the impact of untreated OUD on relationships and ignore the potential role support persons (SPs) could have on encouraging long-term recovery, which can also impact patient treatment retention.

**Methods/design:**

The current study adapts Community Reinforcement and Family Training (CRAFT) for use with SPs (family member, spouse or friend) of patients using buprenorphine/naloxone (buprenorphine) in an outpatient community clinic setting. The study will evaluate whether the adapted intervention, also known as integrating support persons into recovery (INSPIRE), is effective in increasing patient retention on buprenorphine when compared to usual care. We will utilize a two-group randomized design where patients starting or restarting buprenorphine will be screened for support person status and recruited with their support person if eligible. Support persons will be randomly assigned to the INSPIRE intervention, which will consist of 10 rolling group sessions led by two facilitators. Patients and SPs will each be assessed at baseline, 3 months post-baseline, and 12 months post-baseline. Patient electronic medical record data will be collected at six and 12 months post-baseline. We will examine mechanisms of intervention effectiveness and also conduct pre/post-implementation surveys with clinic staff to assess issues that would affect sustainability.

**Discussion:**

Incorporating the patient’s support system may be an important way to improve treatment retention in medication treatments for OUD. If SPs can serve to support patient retention, this study would significantly advance work to help support the delivery of effective treatments that prevent the devastating consequences associated with OUD.

*Trial registration* This study was registered with ClinicalTrials.gov, NCT04239235. Registered 27 January 2020, https://clinicaltrials.gov/ct2/show/NCT04239235.

## Background

Opioid use disorders (OUDs) have profound effects on family members and other support persons (SPs). Hallmark consequences of OUD at the family level include a failure to maintain role obligations at home, important activities given up because of time spent using opioids, continued use despite these problems, and significant impairment in several areas of life [1]. While studies examining the effects of OUD on SPs are sparse, some find that SPs and children of those with OUD experience negative consequences to both physical and mental health, and other quality of life issues [2]. The literature on the effects of substance use disorders on the family is well-documented and provides insights into what families affected by OUD are experiencing. Family members of persons with a substance use disorder are more likely to be diagnosed with depression, substance use disorders, health conditions, or trauma than family members of those with other chronic conditions such as diabetes or asthma. The financial burden is also high, with financial consequences associated with job loss in caring for their person, and higher healthcare costs and utilization associated with their own health and the health of their person [3].

Treatment for OUD is underutilized. Several medications, such as methadone, extended-release injectable naltrexone, and buprenorphine/naloxone (buprenorphine) are efficacious for treating OUDs and are appropriate for delivery in medical settings. Buprenorphine has several advantages over other medications for OUD because it is a feasible and effective treatment to initiate and manage OUD in primary care settings, while also decreasing craving for opioids. Despite the effectiveness of these medications and their potential for helping to combat the opioid crisis [4], only one in five people with an OUD receives medications for OUD. Additionally, standard treatment retention rates for primary care patients receiving buprenorphine for OUD in community settings are suboptimal, with approximately half of patients dropping out within the first 12 months of initiating treatment [5–7].

Involvement of SPs in treatment of people with OUD may prevent premature buprenorphine drop out [8]. A SP can be defined loosely as a partner, parent, other family member or close friend. The SP can be an important catalyst for engaging persons into substance use treatment because SPs are more likely to recognize warning signs of substance misuse compared to the affected individual, who may not recognize or acknowledge symptoms [9, 10]. SPs tend to be highly motivated and typically want to help their person reduce their substance use, improve their relationship, and also alleviate their own difficulties associated with their person’s substance use [11, 12]. On the other hand, SPs can perpetuate their person’s use when they unknowingly facilitate their person’s substance use, fail to recognize or reinforce their person’s steps towards recovery, or are a barrier to their person accessing effective treatments [13, 14]. Further, SPs often have concerns about their person using pharmacotherapies long term and the safety of opioid agonist therapies. In fact, a common barrier to buprenorphine retention is the assumption that buprenorphine is an addictive drug substitute [15]. Thus, engaging the SP as part of the person’s treatment process could be a crucial part of the person’s engagement with and retention in treatment.

The Community Reinforcement and Family Training (CRAFT) intervention was developed to teach the SP how to engage a treatment-resistant person into treatment through positive communication and other behavioral strategies [16, 17]. Instead of “tough love” or encouragement to “detach” from the substance user, CRAFT takes an alternate approach that emphasizes SP empathy and support. CRAFT teaches the SP supportive and non-confrontational skills to improve the relationship between the SP and person (e.g., positive communication, pleasant activity planning), and ways to recognize and reinforce their person’s behaviors that are aligned with treatment goals and recovery (e.g., rewards for sobriety; allowing their person to experience naturally occurring consequences of substance use). CRAFT utilizes functional analysis so that SPs learn about the context around their person’s substance use (e.g., patterns, triggers, rewards) and offers support for how SPs can help reduce their person’s substance use and encourage help-seeking. CRAFT has been adapted for a variety of disadvantaged populations in the U.S. and internationally in six countries [18].

CRAFT is typically composed of 12 individualized sessions that focus on positive communication and other behavioral strategies to help the SP influence their person’s drinking or drug use, and change their negative interactions [16, 17]. Studies show that when an SP receives individualized CRAFT, their person is two to three times more likely to initiate alcohol or drug treatment within 6 months compared to SPs who attend Johnson or 12-step interventions [19–24]. SPs in CRAFT also report improvements in their depression, anger expression toward partner, relationship satisfaction, and family conflict. Few studies have examined group-based CRAFT, but of those, the level of engagement into substance use treatment is similar when compared to self-guided CRAFT [20].

Research supports the effectiveness of CRAFT for treatment initiation but is lacking in two ways: [1] examination of treatment retention outcomes, and [2] application to OUD. Only a single study found that CRAFT can improve treatment retention and drug use outcomes for adults with OUD in specialty care settings compared to treatment-as-usual [8]. The proposed research adapts CRAFT for SPs of patients already engaged in buprenorphine treatment (Phase 1) and examines the effectiveness of the adapted CRAFT intervention (called INSPIRE) on patient buprenorphine retention (Phase 3). We also examine staff and clinic-level process factors that may influence implementation, effectiveness, and sustainability of the intervention in Phase 2. Our study will be the first to evaluate the impact of the intervention on both SP and patient outcome measures within the context of OUD in community health clinics (CHCs).

## Specific aims and hypotheses

The Specific Aims of this project are to: [1] assess the effectiveness of INSPIRE compared to usual care (UC) on buprenorphine retention (primary outcome); [2] examine which patient sub-populations (e.g. addiction severity, race/ethnicity, SP relationship type—e.g., parents, spouse) benefit most from SP involvement in INSPIRE; and [3] assess the patient, provider, and clinic-level factors thought to influence implementation, effectiveness, and sustainability of buprenorphine and INSPIRE. We hypothesize that patients with SPs participating in INSPIRE will have greater buprenorphine retention than patients whose SPs are not participating in INSPIRE, and, consistent with existing CRAFT trials, SPs will experience improved health and quality of life [19–29].

## Methods/design

### Overview of study procedures

All procedures have been approved by an Institutional Review Board and will be renewed annually. There are three phases to the study. Phase 1 will be to adapt CRAFT for SPs of patients currently taking buprenorphine, to include content specific to buprenorphine retention and OUD. We will conduct separate focus groups with patients currently on buprenorphine and SPs affected by OUD. Phase 2 will be a clinic staff survey to assess staff acceptability of buprenorphine and INSPIRE (e.g., fit with current practices, benefit to providers and patients, effectiveness, motivation/willingness to support the intervention, attitudes toward patients with OUD) administered before and after INSPIRE is implemented. Phase 3 will be a two-arm randomized control trial (RCT) of the adapted 10-group INSPIRE intervention compared to UC. Potential patients will be screened upon starting or restarting buprenorphine at one of 12 CHCs. If patients are eligible and have an SP who agrees to participate in the study, those SPs will be randomized to either INSPIRE or UC. Because the focus of the intervention is on the SP, the study will not modify the clinical care of patients receiving buprenorphine.

### Study setting

This study will take place in 12 CHCs in southern and northern California that integrate primary care and behavioral health services in areas of service need. The selected CHCs serve predominantly low-income individuals that qualify for Medical. These 12 CHCs belong to one of three larger health systems that already have established buprenorphine clinics. Some clinics have group refill models and conduct inductions at home, while other clinics prescribe buprenorphine to patients individually and conduct office-based inductions. All patients receiving buprenorphine do so on an outpatient basis. Typically, an individual interested in receiving buprenorphine calls a central phone number to schedule a phone intake with a clinic staff person who will proceed with home induction instructions and/or schedule the individual for an in-person appointment with an X-waivered provider.

### Participants

In Phase 1, eligible participants will be patients 18 or older who are currently receiving buprenorphine and nominated to participate by one of their buprenorphine treatment providers. Eligible SPs will be 18 or older nominated by patients to participate. In Phase 2, staff will include all medical staff who interface with patients in buprenorphine treatment. In Phase 3, eligible study participants will be patients 18 or older who: [1] have completed at least one medication evaluation visit; [2] have no medical contra-indications to buprenorphine, as determined by the patient’s provider; and [3] have an eligible SP they are willing to have participate. Patients who are new to buprenorphine (defined as [1] not receiving a buprenorphine prescription from the study clinic in the past 90 days and [2] patients who return to buprenorphine, defined as not taking buprenorphine for seven or more days in the past 30 days) will be screened. Eligible SPs are those who are: [1] 18 or older; [2] in frequent contact with their person (three times a week or more); [3] not concerned they would be physically hurt by their person; [4] willing and available to attend sessions to address issues related to opioid use by their person if assigned to INSPIRE; [5] committed to their relationship in the next 90 days (no plans to move or end the relationship), and [6] do not currently have a problem with heroin or pain pills [21, 23]. To enroll, the patient’s SP must consent within 1 month of patient consent; this timing was determined based on feedback from providers that some SPs are reluctant to be a part of the patient’s recovery until they see them start buprenorphine. Patients and SPs must both consent for the dyad to be enrolled. According to data from the 12 CHCs, on average patients on buprenorphine were about 51% male; 62% White, 18.2% Multi-race, 13% Black/African American, 3.7% Asian/Pacific Islander, and 2.7% American Indian/Alaskan Native. Research staff will consent individuals.

Participants will be largely low-income and living in urban/suburban areas. While the clinics serve predominantly ethnic minority patients, the demographics of patients that access buprenorphine are more commonly low-income and uninsured White males. These demographics are consistent with the literature showing that rates of OUD are highest among low-income and uninsured individuals [30–32] and that the demographics of patients receiving buprenorphine in federally licensed opioid treatment programs are 69% White and 50% unemployed [33].

### Description of INSPIRE

**Fig. 1:**
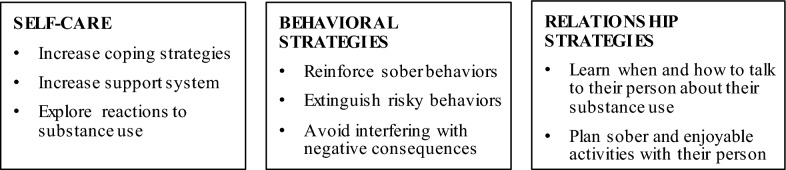
Core functions of CRFAT

INSPIRE consists of ten 90-minute group sessions with SPs (see Table 1). Sessions will be open to allow new SPs to join at any session (“rolling admission”), improving feasibility and sustainability. Sessions are co-led by two medical or behavioral health staff members with knowledge and experience in cognitive behavioral therapy and/or motivational interviewing. Group facilitators are encouraged to offer group participants genuine affirmations throughout sessions. INSPIRE retains important elements of CRAFT that teach the SP supportive skills to cope with their person’s substance use (e.g., positive communication, pleasant activity planning), and ways to interact with their partner through behavioral strategies (e.g., rewards for sobriety, letting natural consequences occur from substance use; see Fig. 1). The sessions also utilize functional analysis or roadmaps so that SPs learn about the context around their person’s substance use (e.g., patterns, triggers, rewards) and offers support for how SPs can help reduce their person’s substance use and encourage help-seeking. The goals of the INSPIRE sessions will be to teach SPs strategies to help their person reduce/refrain from using substances. It focuses on improving the lives of both the SPs and the individuals struggling with substance use.

**Table 1:** Proposed INSPIRE sessions

Session	Session topic
1	Problems due to opioid use
2	Positive rewards
3	Communication: timing and understanding
4	Increasing social support and positive activities
5	Buprenorphine psychoeducation
6	Responding to problem behaviors
7	Roadmap of opioid use
8	Communication: open-ended questions, affirmations, reflections
9	Symptoms and self-care
10	Naloxone and relapse

Each session begins with a welcome and overview that reviews the guidelines for the group. Then, facilitators review practice assignments from the prior session, discuss the session topic, and wrap up with practice for the next week. The sessions are interactive, encouraging participants to practice skills using role plays, pair shares, and other group exercises. Because each unit is self-contained and includes a review of the rationale and skill guidelines, the group can have rolling admission. Rolling groups are common and more sustainable than closed groups in community settings [34–39], which makes the proposed INSPIRE groups comparable to UC.

## Procedures

### Phase 1

The purpose of Phase 1 is to adapt CRAFT to be suitable for CHCs serving patients already engaged in buprenorphine treatment and to receive feedback from patients, SPs, and other stakeholders to determine if INSPIRE is appropriate and helpful.

#### Involving stakeholders in the adaptation

In Phase 1, we will ensure that the INSPIRE intervention is relevant for diverse SPs and feasible to administer as group sessions within the CHC setting. We will follow Barrera and Castro’s [40] framework, which includes information gathering from key informants (i.e., patients, SPs, counselors, CHC administrators), preliminary adaptation of the INSPIRE manual, pilot testing using SP focus groups, and further revisions incorporating feedback from the focus groups. Our goal is to ensure that INSPIRE is compatible for SPs of patients already in buprenorphine treatment, and that we maintain fidelity to the core elements of CRAFT.

First, we will engage a patient and SP stakeholder panel, which consists of patients and SPs who have been and/or are currently affected by OUD and other substance use disorders. They are intended to represent the population of interest on this project but are currently not in treatment (to avoid dual roles). The patients are from the CHCs participating in the study and have longstanding relationships with the clinic as peer navigators and/or serve on the clinic’s board of directors to advocate on behalf of other patients. The SPs are parents and partners affected by OUD, some of whom have experienced tragic loss from OUD and others who have utilized CRAFT with their family members. Several of the SPs have established foundations to help other families affected by substance use. We will also gather input and feedback from a clinic stakeholder panel, composed of medical and behavioral health providers at each of the participating clinics, who actively serve patients on buprenorphine and provide services to their SPs. CHC administrators are also included. Both stakeholder panels will meet once a month.

Finally, we will gather information by conducting two focus groups, one with patients and one with SPs. These focus groups will each consist of 8-10 existing patients and SPs recruited from one CHC. The focus groups will elicit general reactions about INSPIRE, potential session topics, and important adaptations we need to account for when adapting the INSPIRE intervention (e.g., what psychoeducation about buprenorphine and OUD would SPs want to know about?). During the SP focus group, we will demonstrate parts of the INSPIRE sessions and then ask SPs for their feedback. Our final step in the intervention adaptation process will be to revise our manual to incorporate suggestions commonly brought up across the stakeholder meetings and focus groups.

#### Focus group recruitment

We will recruit patients at the participating CHCs who are already in buprenorphine treatment because we want feedback about their experiences with their SP as they started medication; we also want their opinions regarding recruitment procedures for the randomized trial. Patients will be recruited at medication refill groups by case managers and counselors who will briefly describe the focus group and pass out a flyer with RAND’s contact information and instructions how to sign up for the group. Patients are also asked for nominations of SPs who would be interested in a SP focus group. Individuals who attend the two-hour focus group will receive $50 remuneration.

##### Phase 1 analysis plan

Focus groups will be audio recorded. Following grounded theory analyses [41], we will discuss each category and generate underlying themes. Classic content analysis will be used to identify quotes that fit each theme [42, 43]. Then, we will sort quotes by theme and reach a consensus on any discrepancies. This analysis will allow us to understand feasibility and acceptability and will inform the delivery of INSPIRE in a diverse CHC setting.

### Phase 2

The purpose of Phase 2 is to assess staff and clinic-level process factors thought to influence implementation, effectiveness, and sustainability of INSPIRE. Assessing contextual factors that could affect implementation is important for two reasons. First, it will help us interpret differences in intervention effects (if any) between clinics. Second, it will help increase the speed of translation of the INSPIRE intervention into practice, by informing further adaptation of the intervention protocol and by identifying provider factors that could impede or facilitate future implementation.

We will conduct two surveys with study staff at each clinic (pre- and post-RCT) to assess staff acceptability of buprenorphine and INSPIRE. The survey will include questions related to the implementation of INSPIRE including its acceptability, ease of use, fit with current practices, provider motivation and willingness to implement new practices, and attitudes. These questions are based on organizational theory suggesting associations between these factors and successful and sustained implementation of new practices within organizations [44, 45]. Attitudes about people with OUDs, such as stigma, and about buprenorphine in general may also affect how buprenorphine treatment and INSPIRE are implemented [46, 47]. We assess these factors at two time points because participation in the study could change attitudes about buprenorphine and INSPIRE over time, and changes in attitudes could affect variation in effects as well as the likelihood of sustainability, if effective. We will also conduct interviews with providers, patients, and SPs who participate in INSPIRE (post-RCT only) to assess acceptability of INSPIRE and of receiving INSPIRE through CHCs, and factors that could improve the intervention.

#### Staff recruitment

Participants will be medical and behavioral health providers working in the 12 CHCs who treat individuals with OUD. These include staff of varying clinic backgrounds ranging from medical assistants, nurses, psychologists, and physicians from primary care and/or the buprenorphine clinics. We will obtain staff lists and email addresses from each clinic. We will use a combination of in-person and web-based surveys. Where possible, we will distribute surveys at staff meetings in person. Surveys will also be emailed to providers via a web link. Mixed-modes are often used in survey research to meet the challenge of declining response rates, coverage problems in single-mode surveys and the development of web surveys [48]. Using these two modes (web survey and paper/pen) from prior surveys in a primary care study, along with a series of email reminders, we achieved an overall response rate across all staff members of 84–90% across four time points [49]. Providers will be offered the opportunity to win a $100 gift card, with three to four winners from each clinic drawn after each wave. Surveys will be conducted in year 1 prior to the RCT, and in year 4, after the RCT.

##### Phase 2 analysis plan

We will conduct analyses of contextual factors from the provider survey data to help put into context the findings of the RCT, particularly if we observe differences in outcomes across sites. We will conduct bivariate tests of whether provider attitudes at baseline and at follow-up significantly differ across sites. We will examine whether contextual factors and provider attitudes are associated with differences in patient outcomes between sites. Our exploratory hypotheses are that clinics with higher organizational context scores (e.g., less burnout, greater acceptability of new practices, more positive attitudes towards people with OUD) will have greater retention of patients in OUD treatment than clinics with lower scores.

### Phase 3

**Fig. 2:**
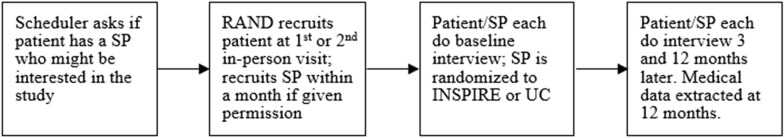
Study flow

The purpose of Phase 3 is to determine the effectiveness of a 10-session INSPIRE intervention compared to UC on patient buprenorphine retention. Recruitment will be conducted through the 12 CHCs. Patients who wish to initiate buprenorphine call the CHCs appointment scheduler, who will schedule the patient for an initial evaluation with an X-waivered medical provider (see Fig. 2). Medical providers will assess the patient’s eligibility for buprenorphine and if they do not present with any medical complications, the patient will be eligible for the study. The medical provider or other clinic staff will then ask whether the patient has a SP that lives locally and with whom they are in frequent contact. If the patient reports having an SP, the staff person will ask whether the SP currently has a heroin or pain pill problem. If the patient reports the SP does not have problems with opioids, the clinic staff member will obtain the patient’s consent to provide the patient’s contact information to RAND and will inform RAND staff of a potential eligible patient.

RAND will contact the patient and describe the study in more detail. A RAND study team member will meet patients in person at the clinic, however, if in-person contact is not feasible, this procedure could occur by phone. RAND staff will ask patients for permission to contact their SP. The goal would be to identify SPs who have a significant impact on their life [50]. RAND will recruit the patient at their first or second visit with the medical provider as some patients may be in withdrawal at their first visit. SPs will be recruited within a month of the patient’s consent.

**Fig. 3:**
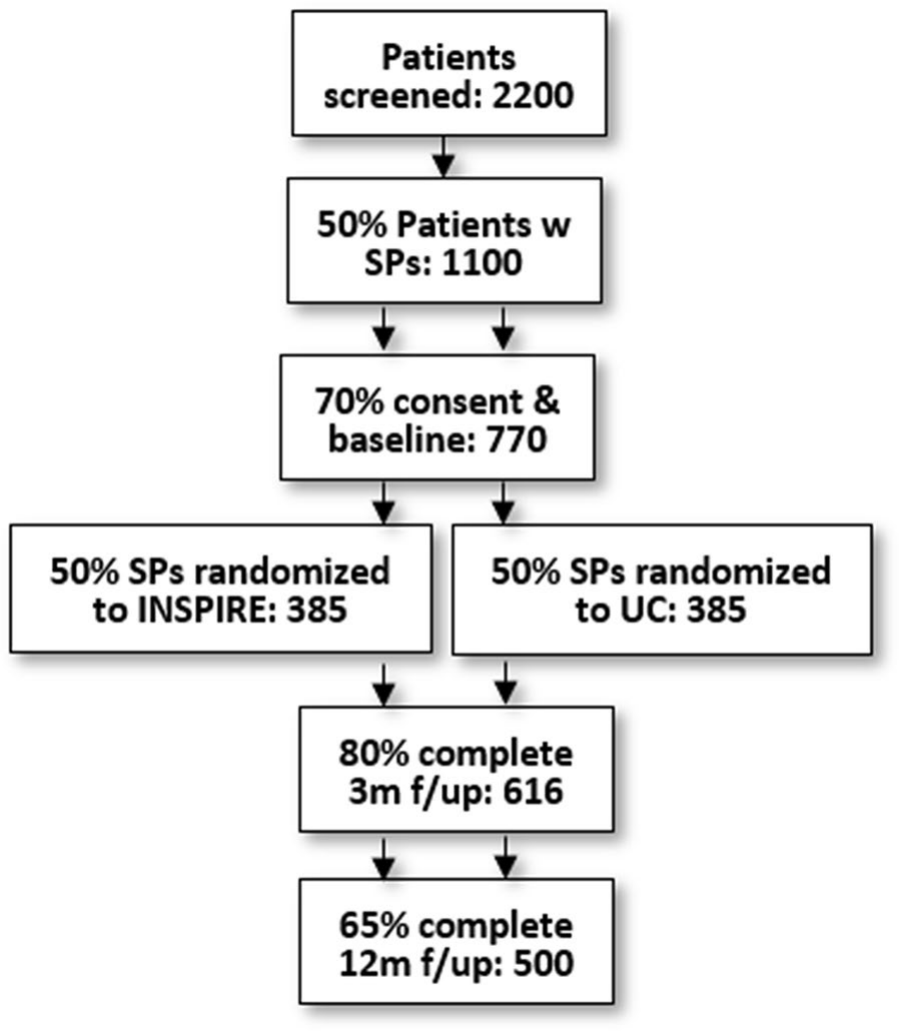
Study flow

Once enrolled in the study, patients and SPs will be interviewed separately at all time points (baseline, 3, 12 months). Participants will receive a $5 incentive for screening, $30 for the baseline interview survey, $30 for the 3-month interview and $40 for the 12-month interview. In addition, SPs assigned to INSPIRE may receive transportation and/or childcare remuneration to attend sessions as needed. All participants and SPs will be contacted for follow-up, regardless of whether they complete INSPIRE or UC, and we will conduct intent-to-treat analyses. A total of 770 dyads are expected to consent to the study, resulting in a total of 616 dyads at 3-month follow-up (assuming 80% retention) and 500 dyads at 12-month follow-up (assuming 65% retention) (see Fig. 3). To ensure robust follow-up rates, we will obtain detailed information at baseline on how to reach participants and use proven methods to minimize attrition, including an in-person baseline interview to build rapport, obtaining multiple contacts (friends/families/service providers) at baseline for individuals who would know the participants’ whereabouts, and phone/mail/text/social media reminders prior to follow-up.

We will examine patient electronic medical records (EMRs) to assess buprenorphine medication retention (i.e., no more than a 7-day lapse between prescriptions) [51, 52], healthcare utilization (e.g., number of medical and behavioral health visits), and results of urine tests (e.g., presence of buprenorphine and other opioids). All data will be stored on encrypted laptops used only for research purposes that require staff to login and provide a personal password to access. Files that have personally identified health information will also be password protected.

Randomization will conducted by research staff at the dyad-level such that patients and SPs both need to consent to be randomized. Dyads will be stratified by clinic and by SP type. We will stratify participating SPs who are patients’ parents equally to INSPIRE or UC because of existing CRAFT studies showing a stronger effect of CRAFT for parents [8] using permuted block randomization with random size blocks. This ensures the number of people allocated to each group is approximately equal throughout recruitment [53]. Data collectors will not be blind to SP intervention condition because they will need to schedule SPs for the INSPIRE class, but are independent and not part of the core research team. In addition, to minimize bias, they will emphasize to participants that their responses would be confidential and not conveyed to the clinics, will conduct follow-up interviews outside of the clinic, and will build rapport with participants to increase self-report validity.

#### INSPIRE facilitators and training

Medical and behavioral health staff members who will be facilitating INSPIRE groups (selected nurses, case managers, peer recovery specialists, and licensed social workers) from each CHC will attend a 1-day training, conducted by the study team. The training will involve a mix of didactic presentation, demonstration, and practice sessions involving role-plays. Role-plays are central to practicing INSPIRE and are highlighted throughout the training to give facilitators the opportunity to practice and receive feedback. The training will focus on group facilitation skills, safety planning, enhancing SP motivation to engage in INSPIRE, and the importance of helping the SP to increase positive interactions between the SP and patient. Key techniques, such as communication and recognizing and reinforcing positive behaviors, will be taught. Skills also include ways to reinforce and shape patient behaviors with an emphasis on refraining from interfering in naturally occurring OUD consequences. The training will focus on ways the SPs can respond to problematic patient behaviors, as well as problem-solving and ways to facilitate patients’ retention in buprenorphine. Staff will receive weekly supervision thereafter and will be monitored using a fidelity checklist.

#### INSPIRE adherence monitoring and supervision

We will use methods from our previous studies to assess adherence to session content [54, 55]. A basic checklist will be developed outlining each session’s content. All sessions will be audio recorded and files will be uploaded by facilitators to a secure web server. Session recordings will be reviewed and coded by the INSPIRE trainer and supervisor who will code whether content was discussed and rate the facilitators’ proficiency discussing the content. Weekly phone supervision will also take place where experiences from the prior group are discussed, feedback from recordings provided, and review of next session’s content is outlined and practiced. The intervention will be modified or stopped as determined by the DSMB and IRB if there is clear evidence of harm (e.g., worsening symptoms), a favorable benefit:risk ratio, or an unfavorable risk:benefit ratio. All adverse events will be recorded and communicated to the sponsor, DSMB and IRB per our Data Safety Monitoring Plan protocol. Any modifications to the protocol will be reported to relevant parties including the investigators, participants, sponsor, IRB, and clinicaltrials.gov where the study is registered.

#### Usual care

All patients will be receiving buprenorphine as part of usual procedures in the clinic. Services available for SPs vary by clinic and may include individual behavioral health counseling, psychoeducation groups, and standard primary care services. We will collect information from support persons in UC about the services they received to further quantify this condition.

#### Outcomes

Our primary outcome is patient retention on buprenorphine, measured using data from the EMR, defined as buprenorphine prescriptions of at least 6 and 12 months with no more than a 7-day lapse between prescriptions (see Table 2) [8, 51, 52, 56]. We have two sets of secondary outcomes that correspond to the effectiveness of INSPIRE (specific aim 1). For the patient, we will validate adherence using urine test results in the EMR for buprenorphine metabolites, and will assess, through patient self-report, patient opioid and other substance use in the past month, overdose risk, substance use problems, health, other employment, quality of life, communication, employment, and treatment satisfaction. For the SP, our secondary outcomes are self-reported and will include the SP’s physical and mental health (depression and anxiety), substance use, quality of life, and relationship quality with their person. These outcomes have been validated with high psychometric properties in existing CRAFT trials [8, 19–24]. Self-report will be assessed via in-person interview during baseline, and phone-based interviews at 3- and 12-month follow-ups. RAND staff who conduct interviews will be masked to condition.

**Table 2:** Outcome measures

Source	Timepoint	Outcome	Description
Primary outcome measures			
EMR	6, 12	Buprenorphine retention	Percentage of patients who initiated buprenorphine who have at least 6 and 12 months of continuous treatment with buprenorphine with no more than a 7-day lapse between prescriptions [51, 52] Number of days of longest continuous buprenorphine treatment with no breaks in care

Source		Outcome	Description
Secondary patient outcome measures			
P INT SP INT	BL, 3, 12 BL, 3, 12	Patient opioid use	Heroin, prescription opioid, and other opioid use in the past 30 days using questions from the National Survey on Drug Use and Health [57–59]
EMR	6, 12	Medication adherence	Percentage of patients with a buprenorphine prescription with a urine test positive for buprenorphine metabolites
EMR	6, 12	OUD treatment retention	Percentage of patients who initiated OBOT who had continued visits (no more than a 30-day lapse between visits) with any provider for a substance use disorder for 3, 6, and 12 months [8, 56]
P INT SP INT	BL, 3, 12 BL, 3, 12	Patient other substance use	Alcohol and other drug use in the past 30 days [58, 59]
P INT	BL, 3, 12	Overdose risk	Opioid Overdose Risk Assessment scale [60]
P INT	BL, 3, 12	Patient Substance use problems	Substance-related problems in the past 30 days using the Short Inventory of Problems
P INT	BL, 3, 12	Patient health	Physical health in the past 30 days; depression and anxiety
P INT	BL, 3, 12	Quality of life	Functional impairment in work/school, family, and social life using the 5-item Sheehan Disability Scale [61]
P INT	BL, 3, 12	Communication	Adapted from the FACES IV [62]
P INT	BL, 3, 12	Employment status	Full-time, part-time, unemployed, other (e.g., students, persons keeping house or caring for children full time, retired or disabled persons) [63]
EMR P INT	6, 12 BL, 3, 12	Healthcare utilization	Number of outpatient medical and behavioral health visits over the 12-month period
P INT	3, 12	Treatment satisfaction	Client Satisfaction Questionnaire (CSQ-8) [64]
Secondary SP outcome measures			
SP INT	BL, 3, 12	Health	Physical health in the past 30 days; depression and anxiety
SP INT	BL, 3, 12	Substance use	Alcohol and other drug use in the past 30 days [58, 59]
SP INT	BL, 3, 12	Quality of life	Functional impairment in work/school, family, and social life using the 5-item Sheehan Disability Scale [61]
SP INT	BL, 3, 12	Communication	Adapted from the FACES IV [62]
SP INT	3	INSPIRE satisfaction	Client Satisfaction Questionnaire (CSQ-8) adapted to INSPIRE [64]

BL: baseline; 3: 3-month follow-up; 6: 6-month; 12: 12-month follow-up; EMR: electronic medical record; P INT: patient interview; SP INT: support person interview; FACES IV: Family Adaptability and Cohesion Scale IV; OBOT: office-based opioid treatment

##### Phase 3 sample size and power

We focus our power calculations for the pre-specified comparative effectiveness analyses on the primary outcome of retention at 12 months, for which we will have 250 dyads per condition (N = 500 dyads total). Based on literature, we assume 51% of patients in OBOT will be retained in treatment [65]. We would have 80% power (alpha = 0.05/2, two-sided, two-sample test) to detect a difference of 51% versus 65% between OBOT and CRAFT + OBOT. Our ability to detect this effect is plausible, considering Roozen et al. [23] reported an even larger difference of 30% versus 64% in a systematic review of CRAFT for substance abuse treatment. Given this primary statistical test and the pre-specified heterogeneity of treatment effect (HTE) analysis examining retention described below, we adjust for multiple comparisons by using an alpha level of 0.05/2. To further account for multiple statistical testing given that we will also conduct secondary analyses, we will compute and report false discovery rates [66].

##### Phase 3 analysis plan

Our data monitoring team consist of a programming analyst and statistician who work at RAND and are independent of competing interests. The statistician will supervise the programmer in analyses and will convey to the core research team their analytic process and findings. Specifically, they will use generalized linear mixed models to compare primary and secondary outcomes for patients with SPs in INSPIRE versus UC at 3- and 12-month follow-ups. They will also examine patient treatment retention at 6 and 12 months using medical records. They will include baseline characteristics in the models and do not plan to link 3-month interview data to retention outcomes, thereby allowing us to examine short-, medium-, and long-term outcomes in this study. Fixed effects for clinic and SP type (parent vs. other) will be included as covariates to account for the stratified study design [67] and random intercept terms to model correlation among repeated measures within patient, while controlling for additional patient characteristics (age, gender, race/ethnicity, addiction severity). They will modify the model accordingly if diagnostics indicate the model assumptions are violated. To handle missing data, we may use covariate adjustment and weighting, assuming data are missing at random and multiple imputation. Sensitivity of results to missing data will be assessed by examining whether baseline characteristics of the sample, including treatment assignment, differ for study completers versus drop-outs.

Findings will be disseminated at professional conferences and scientific manuscript publication. Data sharing agreements may be possible after the main project findings are accepted for publication.

## Discussion

The proposed research fills at least four gaps. First, buprenorphine retention is poor and this negatively impacts OUD treatment outcomes. The proposed research examines whether group-based INSPIRE helps retain patients in primary-care-based buprenorphine treatment. To date, only one small study (N = 52 dyads) has examined individual-based CRAFT for OUD during the transition between opioid detoxification and specialty outpatient treatment. The study found moderate effects on treatment retention and patient opioid and other drug use 9 months later [8]. Our study will be the first to evaluate 12-month retention outcomes for patients receiving buprenorphine in CHCs where primary care and behavioral health are integrated. Our study has the advantage of measuring long-term retention through EMRs, which existing CRAFT trials have not done. Second, CRAFT has been evaluated mostly in the community where SPs are recruited through local advertisements; this will be the first study to evaluate an adapted CRAFT intervention in CHCs that serve predominantly low-income populations. Third, we evaluate INSPIRE on both patient and SP outcomes, and whether SP relationship type (e.g., parent vs. other) affects patient outcomes. This fills research gaps because most existing CRAFT trials measure patient outcomes through SP, do not evaluate long-term SP outcomes, and do not stratify recruitment by SP relationship type. Finally, our study will be the first to evaluate the impact of INSPIRE on both SP and patient outcome measures of physical and mental health, quality of life, and relationship quality. The proposed study not only fills these critical gaps in the literature, but also provides data to inform a much-needed new way to address the opioid epidemic.

## Data Availability

Not applicable.

## Abbreviations

CHC: Community health clinic
CRAFT: Community Reinforcement and Family Training
EMR: Electronic medical record
FACES IV: Family Adaptability and Cohesion Scale IV
INSPIRE: Integrating support persons into recovery
OUD: Opioid use disorder
P INT: Patient interview
RCT: Randomized control trial
SP: Support person
SP INT: Support person interview
UC: Usual care
OBOT: Office-based opioid treatment
